# A comparative study of the degradation of yeast cyclins Cln1 and Cln2

**DOI:** 10.1002/2211-5463.12157

**Published:** 2016-12-14

**Authors:** Inma Quilis, J. Carlos Igual

**Affiliations:** ^1^ Departament de Bioquímica i Biologia Molecular and ERI BiotecMedUniversitat de ValènciaBurjassotSpain

**Keywords:** cell cycle, Cln1, Cln2, cyclin, *Saccharomyces cerevisiae*, SCF ubiquitin ligase

## Abstract

The yeast cyclins Cln1 and Cln2 are very similar in both sequence and function, but some differences in their functionality and localization have been recently described. The control of Cln1 and Cln2 cellular levels is crucial for proper cell cycle initiation. In this work, we analyzed the degradation patterns of Cln1 and Cln2 in order to further investigate the possible differences between them. Both cyclins show the same half‐life but, while Cln2 degradation depends on ubiquitin ligases SCF^G^ ^rr1^ and SCF^C^ ^dc4^, Cln1 is affected only by SCF^G^ ^rr1^. Degradation analysis of chimeric cyclins, constructed by combining fragments from Cln1 and Cln2, identifies the N‐terminal sequence of the proteins as responsible of the cyclin degradation pattern. In particular, the N‐terminal region of Cln2 is required to mediate degradation by SCF^C^ ^dc4^. This region is involved in nuclear import of Cln1 and Cln2, which suggests that differences in degradation may be due to differences in localization. Moreover, a comparison of the cyclins that differ only in the presence of the Cln2 nuclear export signal indicates a greater instability of exported cyclins, thus reinforcing the idea that cyclin stability is influenced by their localization.

**Abbreviations**
CDK: cyclin‐dependent kinase
CKI: cyclin‐dependent kinase inhibitor
CPD: Cdc4 phosphodegron
HA: human influenza hemagglutinin epitope
NLS: nuclear localization signal
PEST: domain rich in proline, glutamic acid, serine, threonine
SCF: Skp1‐cullin‐F‐protein ubiquitin ligase complex

Cell cycle progression is governed by the sequential activation of different cyclin‐dependent kinase (CDK) complexes. For the yeast *Saccharomyces cerevisiae*, nine different cyclins (Cln1–3 and Clb1–6) activate the Cdc28 protein, the only yeast CDK which performs an essential function in cell cycle progression (reviewed in 1, 2). The level of the associated cyclin and CDK inhibitors (CKI) determines the kinase activities that are present at a given time. Two molecular mechanisms, gene transcription and protein degradation, control these key cell cycle regulators. The coordinated expression of different sets of genes, organized in transcriptional waves along the cell cycle, is a very common strategy in cell cycle control in all eukaryotes. These genes are typically involved in specific cell cycle processes and their maximum expression coincides with the time that their products are required. These transcriptional waves are regulated by transcription factors whose expression is usually also cell cycle regulated in a way that help to organize the waves of expression 3, 4, 5. All the cyclins associated with Cdc28 CDK are periodically expressed. In the case of *CLN1* and *CLN2* genes, both are expressed during the G1/S transition by the transcription factor SBF 6, 7, 8. The second major mechanism involved in the control of the cellular levels of cell cycle regulators is proteolysis by means of ubiquitination and degradation in the proteasome 9, 10, 11, 12. Protein ubiquitination is carried out by the successive action of ubiquitin‐activating (E1), ubiquitin‐conjugating (E2) and ubiquitin‐protein ligase (E3) enzymes 13. Ubiquitin ligases mediate the transfer of the ubiquitin molecule from the E2 enzyme to the ε‐amino of a lysine at the target proteins. Two ubiquitin ligases play an important role in the cell cycle regulation: Skp1‐cullin‐F‐box protein (SCF), which is critical for the G1/S transition and anaphase‐promoting complex (APC), which performs an essential function in mitosis 14, 15, 16.

SCF is involved mainly in the control of the G1/S transition through the degradation of G1 cyclins and CKI 17, 18, 19, 20. However, it can also participate in other cell cycle phases 21. The SCF complex consists in four subunits: Skp1, Cdc53, Rbx1 and an adapter protein with an F‐box. The Rbx1 subunit interacts with the ubiquitin‐conjugating enzyme, Cdc34 in this case, whereas the F‐protein is responsible for substrate recognition. Three F‐proteins have been found to be involved in the degradation of cell cycle regulators: Cdc4, Grr1 and Met30. They present protein–protein interaction domains, such as leucine‐rich repeats LRR for Grr1 or a WD40 domain in the case of Cdc4 and Met30 22. The presence of distinct F‐protein subunits in the complex directs SCF activity to different targets. However, some overlapping sets of substrates may exist since, recently, it has been described that G1 cyclin Cln3 is targeted by both ubiquitin ligases SCF^Cdc4^ and SCF^Grr1^ 23. The other G1 cyclins, Cln1 and Cln2, are also important targets of SCF. They are highly unstable proteins with reported half‐lives under 15 min 20, 24, 25, 26, 27, 28, 29. Cln2 and Cln1 are strongly stabilized in a *grr1* mutant strain 20 and both cyclins bind to Grr1 30. Moreover, the transfer of the C‐terminal region of Cln2 to heterologous proteins confers protein instability mediated by SCF^Grr1^ 31. For this reason, it is assumed that SCF^Grr1^ is the ubiquitin ligase involved in Cln1 and Cln2 degradation. However, Cln1 and Cln2 bind to SCF^Cdc4^ *in vitro* and, in fact, contradictory results have been obtained for the effect of *cdc4* mutation on Cln2 stability 23, 32, 33, 34.

Instability of proteins depends on a degron motif, which is recognized by the ubiquitin ligase. In the case of SCF, recognition by the F‐protein requires the presence of phosphorylated epitopes in the degron. Sic1 has served as a model substrate, so the mechanics of its recognition by Cdc4 are understood in detail 22, 35, 36, 37, 38. This model allowed the description of the consensus Cdc4 phosphodegron (CPD) sites present in SCF^Cdc4^ substrates. The Cdc4 recognition mechanism is highly tunable, and the number and nature of the CPD sites and the targeting kinases 22. The precise Grr1 phosphodegron and how it may differ from the CPD have yet to be precisely defined. PEST motifs (rich in proline, glutamic acid, serine and threonine) have been described in SCF^Grr1^ substrates, such as G1 cyclins Cln1 and Cln2, as being the regions responsible for their instability. In the case of Cln2, deletion of little more than the PEST region (from 373 to 409 amino acids) renders a protein significantly more stable than the wild‐type. Nonetheless, additional stability is achieved by deletions that remove the entire carboxyl terminus (from 373 to 545), indicating that the sequences around the PEST sequence are also involved in the control of Cln2 stability 26. This conclusion is supported by the converse experiment in which the transfer of Cln2^376–545^ confers high instability to a heterologous protein, whereas a smaller region has a milder effect 26, 31. Similarly, a deletion of Cln1 including a portion of its PEST region and its carboxyl terminus (Cln1^266–546^), produces a stable protein 20.

Cln1 and Cln2 are the most similar cyclins in yeast. They show a 57% sequence identity, which increases up to 72% in the N‐terminal region containing the cyclin box domain. The extensive work carried out on these cyclins revealed that both take part in many common functions at the G1/S transition 39, 40, 41, 42, 43, 44, 45, 46, 47, 48. All these results led to the notion that Cln1 and Cln2 are equivalent cyclins. However, in addition to the numerous studies that highlight Cln1 and Cln2 similarities, several functional differences between them have also been described 49, 50, 51, 52, 53. In some cases, the functional distinction seems to be caused by quantitative differences at the expression level. More recently, cyclin‐specific docking motifs in target proteins that bind preferentially to Cln2 54, and a nuclear export mechanism present only in Cln2 that contributes to the functional distinction between Cln1 and Cln2 55, have been described. As regards Cln1,2 degradation, most of the studies have been performed with antibodies that recognize either both cyclins or Cln2 alone, and many of the results reported for Cln2 have been extended to Cln1. To complete a comparative study of Cln1 and Cln2, the present study attempts to analyze the degradation of both cyclins in order to investigate new differences between them.

## Materials and methods

The yeast strains used in this study are W303‐1a (*MATa ade2‐1 trp1‐1 leu2‐3,112 his3‐11,15 ura3‐52*)*, cdc53* ^*ts*^ (*MATa ade2‐1 trp1‐1 leu2‐3,112 his3‐11,15 ura3‐52 cdc53.1*), *cdc4* ^*ts*^ (*MATa ade2‐1 trp1‐1 leu2‐3,112 his3‐11,15 ura3‐52 cdc4‐1*) and JCY1539 (*grr1::LEU2* in W303‐1a). Yeast cells were grown on synthetic dextrose medium supplemented as required. Centromeric plasmids pCLN1 and pCLN2 expressing HA‐tagged versions of Cln1 and Cln2, respectively, were a gift from Dr. M. Aldea. Centromeric plasmids expressing HA‐tagged chimeric cyclins or *CLN1* and *CLN2* genes under the control of the *ADH1* promoter, were described in 55. Centromeric plasmids pNE104 and pNE108 expressing Cln2 fused to a canonical active or inactive NLS, respectively, were a gift from Dr. B. Futcher 56.

To evaluate cyclin stability, 50 μg·mL^−1^ cycloheximide was added to exponentially growing cells or cells incubated during 3 h at 37° as indicated. Samples were harvested at the indicated times and cyclin protein decay was analyzed by western blot as described previously 57. Antibodies used were anti‐HA 3F10 or 12C5A (Roche, Basel, Switzerland), anti‐Cln2 y‐115 (Santa Cruz Biotechnology, Dallas, TX, USA) and anti‐tubulin (Serotec, Bio‐Rad, Hercules, CA, USA). Bands were quantified with an ImageQuant™ LAS 4000mini biomolecular imager (GE Healthcare, Fairfield, CT, USA). At least three independent experiments were carried out for each cyclin/strain. In the case of thermosensitive degradation mutants, although we observed quantitative variations between experiments, the qualitative effect of each mutation in cyclin stability was consistently reproducible; the results shown in figures are representative results of the experiment.

## Results

### Analysis of Cln1 and Cln2 stability

**Figure 1:**
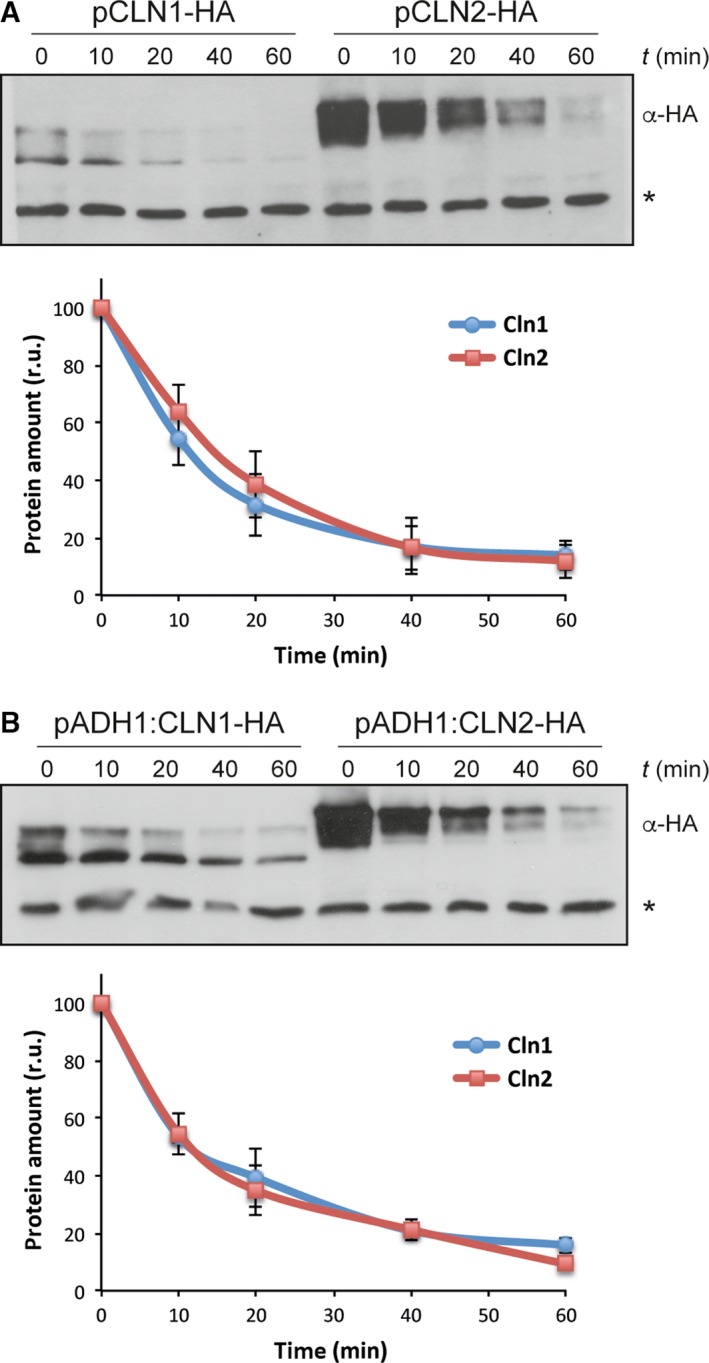
Protein stability of cyclins Cln1 and Cln2. Exponentially growing cells of the wild‐type strain transformed with a centromeric plasmid expressing either a HA‐epitope tagged Cln1 or Cln2 protein at endogenous level (A) or overexpressed from the *ADH1* promoter (B) were incubated in the presence of 50 μg·mL^−1^ cycloheximide. Cln1 and Cln2 protein levels were analyzed at the indicated time after the addition of cycloheximide by western blot. A nonspecific band labeled with an asterisk that cross‐react with the antibody is shown as loading control. Graph represents the relative amount of Cln1 and Cln2 proteins related to the nonspecific band.

To date, no parallel assays of Cln1 and Cln2 protein degradation have been carried out. Therefore, we first compared the stability of the two cyclins separately. Translational shut‐off experiments were carried out, following protein decay after the addition of cycloheximide by western blot. The stability of Cln1 and Cln2 was analyzed in the wild‐type cells expressing HA‐tagged version of the proteins under the control of their own promoters or the *ADH1* promoter (a noncell cycle‐regulated promoter to avoid secondary effects due to cell cycle alterations by cycloheximide treatment). As seen in Fig. 1, both proteins showed identical decay kinetics when expressed endogenously or ectopically. We can conclude that the stability of cyclins Cln1 and Cln2 is similar.

### Ubiquitin ligases involved in the degradation of Cln1 and Cln2

**Figure 2:**
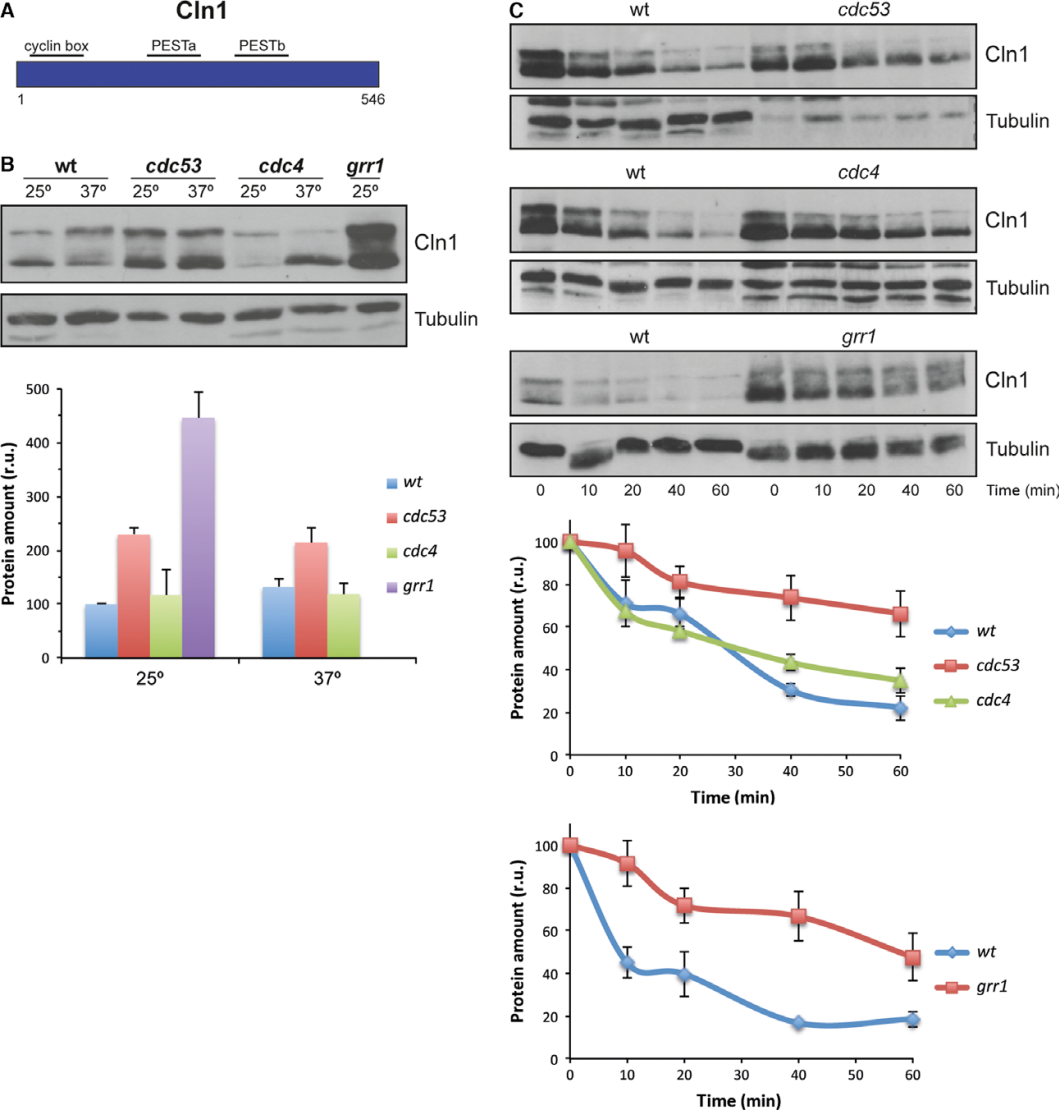
Analysis of Cln1 degradation in SCF ubiquitin‐ligase mutant strains. (A) Scheme of Cln2 cyclin. (B) Cells of the wild‐type (W303), *cdc53*,* cdc4* and *grr1* (JCY1539) strains transformed with a centromeric plasmid expressing a HA‐epitope tagged Cln1 protein at endogenous level were grown at 25° and transferred for 3 h at 37° in the case of *cdc53* and *cdc4* strains. Cln1 protein level was analyzed by western blot. Tubulin is shown as loading control. Graph represents the relative amount of Cln1 protein. (C) Same cells than in B were incubated in the presence of 50 μg·mL^−1^ cycloheximide. Cln1 protein level was analyzed at the indicated time after the addition of cycloheximide by western blot. Tubulin is shown as loading control. Graph represents the relative amount of Cln1 protein.

**Figure 3:**
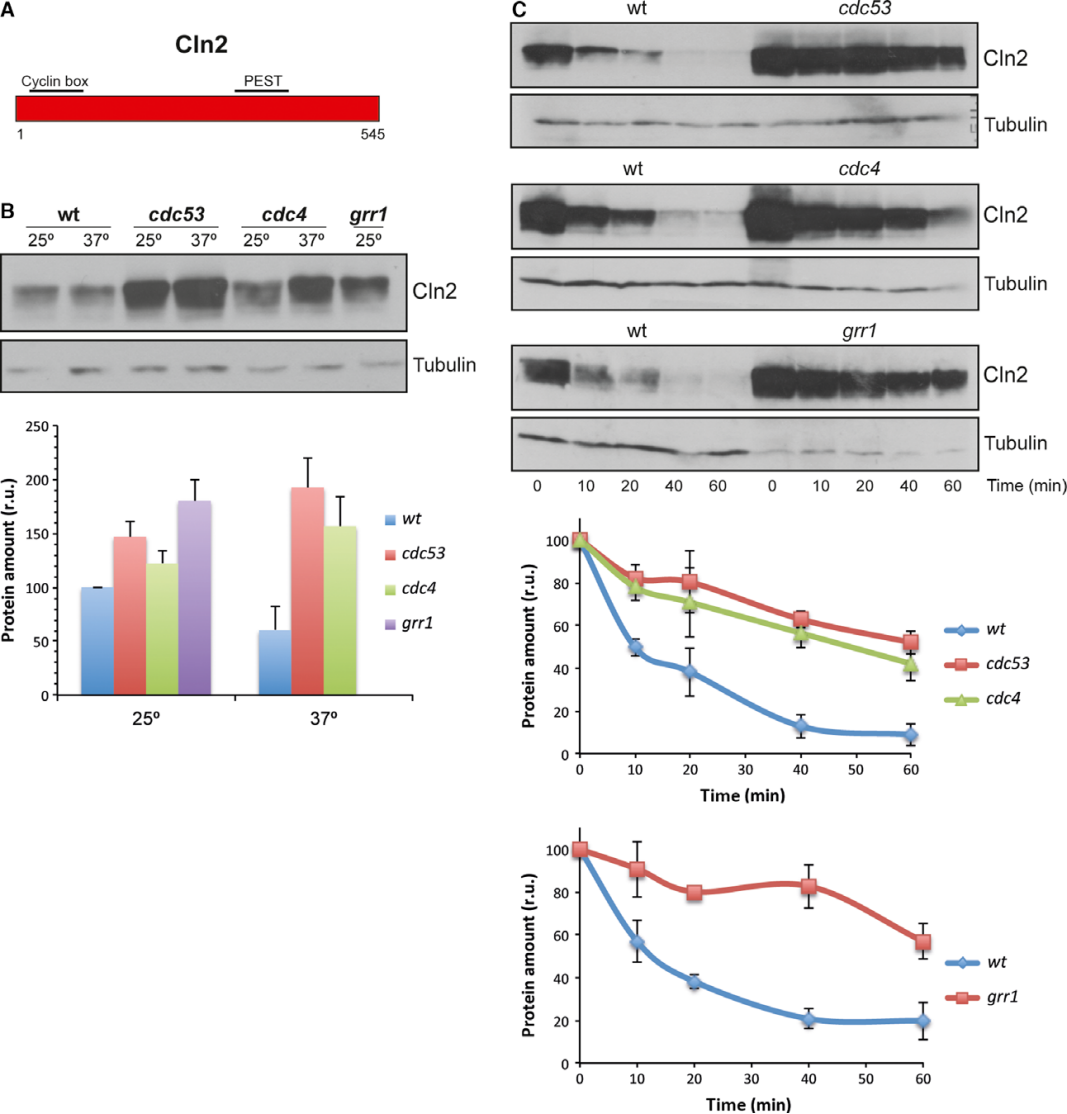
Analysis of Cln2 degradation in SCF ubiquitin‐ligase mutant strains. (A) Scheme of Cln2 cyclin. (B, C) Protein level and degradation of Cln2 cyclin were analyzed as described in Fig. 2.

G1 Cln cyclins are degraded by the ubiquitin ligase SCF pathway. Substrate recognition depends on the F‐box protein of the complex. In the case of cyclins Cln1 and 2, Grr1, but not Cdc4, has been described to be the F‐protein involved in their degradation. However, these experiments jointly analyzed Cln1 and Cln2 or only analyzed Cln2. For this reason, the second aim of this work was to separately study Cln1 and Cln2 stability in mutant strains in the F‐proteins Grr1 and Cdc4 in order to determine whether there are differences in the requirements for a specific F‐protein between both cyclins. Thus, the mutant strains in Cdc4 or Grr1, as well as in the SCF‐cullin subunit Cdc53, were transformed with the plasmids expressing HA‐tagged Cln1 and Cln2, and the cyclin protein levels were compared to those of the wild‐type cells. *CDC4* and *CDC53* are essential genes, so thermosensitive mutant strains were used in these cases. The results obtained are shown in Figs 2 and 3. The Cln1 protein level sharply increased in the *grr1* mutant strain. Importantly, no increase in Cln1 cellular content was detected for the *cdc4* mutant cells at the restrictive temperature, suggesting that only Grr1 is involved in Cln1 degradation. To further investigate this possibility, we also analyzed Cln1 stability in the different mutant strains by translational shut‐off experiments. As shown in Fig. 2C, inactivation of Grr1 but not Cdc4 resulted in a marked stabilization of Cln1. All together, these results indicate that Cln1 is degraded by SCF^Grr1^.

However, a different result was obtained for Cln2. The Cln2 levels increase in the *cdc53* ^*ts*^ and *cdc4* ^*ts*^ mutant strains at the restrictive temperature, as well as in the absence of Grr1 (Fig. 3B), suggesting that both Grr1 and Cdc4 are involved in Cln2 degradation. Consistently with these results, the translational shut‐off assays revealed protein stabilization in the absence of Cdc53, Cdc4 and Grr1 (Fig. 3C). Thus, our results reveal a difference in the degradation of both cyclins: while Cln1 is mostly degraded by SCF^Grr1^, Cln2 degradation depends on both SCF^Cdc4^ and SCF^Grr1^.

### The cyclin regions affecting degradation

**Figure 4:**
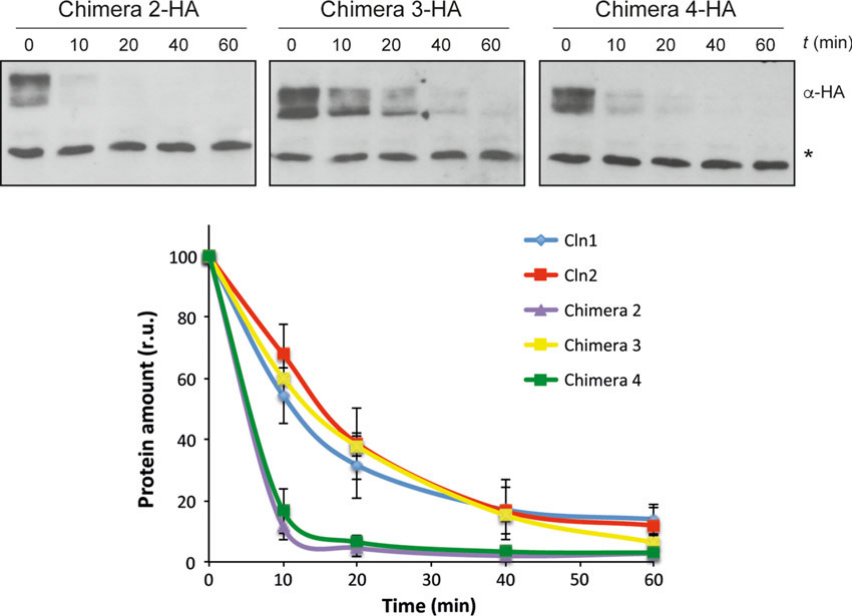
Protein stability of chimeric cyclins. Protein stability of chimeric cyclins was analyzed as described in Fig. 1.

The use of different adaptor proteins in the degradation pathway may be due to different causes, possibly reflecting a difference in the function of the PEST sequences recognized by the F‐proteins. PEST sequences were identified in Cln1 and Cln2 58. These regions were also predicted by the PESTfind program (http://emboss.bioinformatics.nl/cgi-bin/emboss/epestfind). Cln2 contained a PEST sequence between amino acids 371 and 403 with a predicted value of 6.99. Cln1 contained a PEST in a similar region, between amino acids 398 and 424 (PESTb), although it was predicted with a very low value (0.85); another PEST sequence between amino acids 234 and 275 (PESTa) was predicted in Cln1 with a value of 14.12, which was absent in Cln2. In order to determine the relevance of these PEST sequences or additional regions in cyclin degradation, the strategy used consisted in studying the degradation of chimeric cyclins obtained by the exchange of the equivalent regions between Cln1 and Cln2 55. The first question to be addressed was whether the PESTb of Cln1 was functional given its low prediction value. In order to answer this question, we analyzed the degradation of Chimera 2 (Cln2^1–299^‐Cln1^313–546^). This chimera contained the N‐terminal region of Cln2 and the C‐terminal region of Cln1, so the PEST of Cln2 was exchanged for the PESTb of Cln1. The analysis of Chimera 2 stability showed that Chimera 2 was much more unstable than Cln1 and Cln2 (Fig. 4). This result supports the notion that the PESTb of Cln1 was functional and that, in fact, it could prove even more efficient than the corresponding Cln2 PEST.

**Figure 5:**
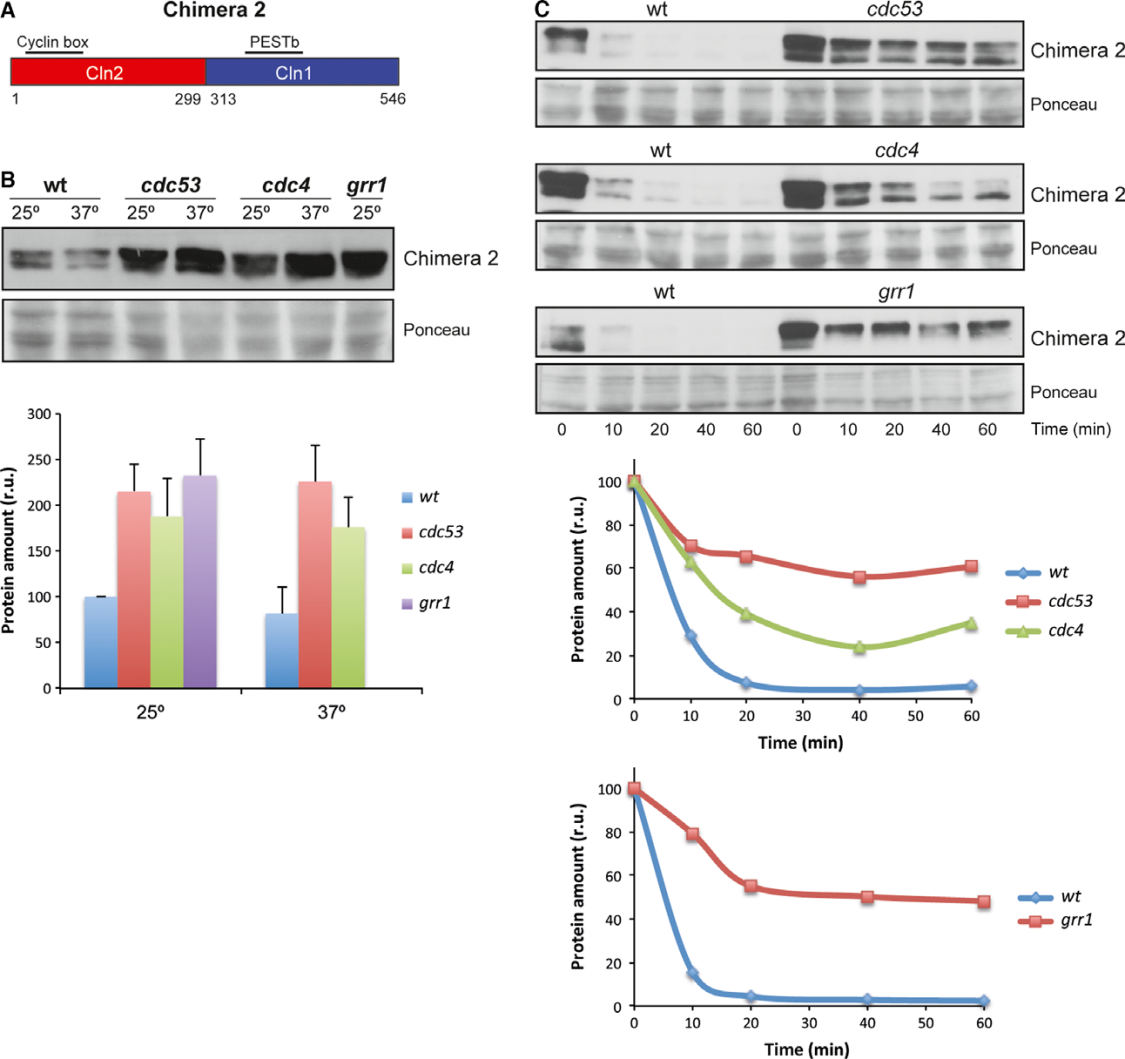
Analysis of Chimera 2 degradation in SCF ubiquitin‐ligase mutant strains. (A) Scheme of Chimera 2 cyclin. (B, C) Protein level and degradation of Chimera 2 cyclin were analyzed as described in Fig. 2. Ponceau staining is shown as loading control.

Next, we tested the protein level and the stability of the chimera in the SCF subunit mutants as described above in order to determine the machinery involved in its degradation. The cellular content of Chimera 2 increased in all the *cdc53* ^*ts*^, *cdc4* ^*ts*^ and *grr1* mutant strains (Fig. 5B). Consistently with this, the translational shut‐off assays exhibited a stabilization of Chimera 2 in all the mutants (Fig. 5C). These results indicate that both SCF^Grr1^ and SCF^Cdc4^ mediated Chimera 2 degradation, similar to that observed for Cln2. The fact that the degradation pattern of Chimera 2 corresponded to Cln2, despite it contains the PESTb of Cln1, indicates that as far as the F‐proteins were concerned, Cln2 PEST and Cln1 PESTb are equivalent and that other factors acting outside the C‐terminal region determined the degradation pattern of the cyclin.

**Figure 6:**
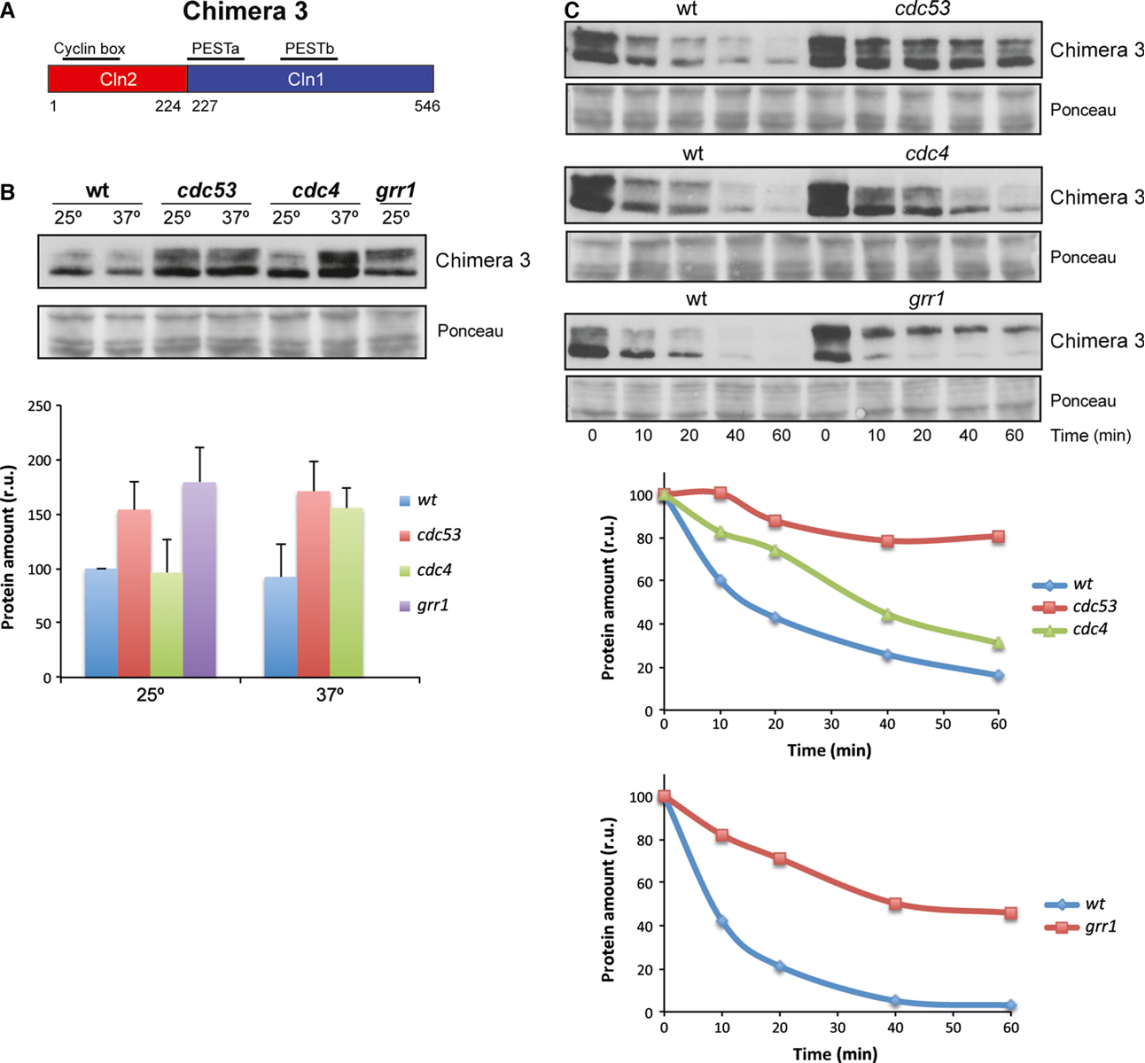
Analysis of Chimera 3 degradation in SCF ubiquitin‐ligase mutant strains. (A) Scheme of Chimera 3 cyclin. (B, C) Protein level and degradation of Chimera 3 cyclin were analyzed as described in Fig. 2. Ponceau staining is shown as loading control.

We also analyzed Chimera 3 (Cln2^1–224^‐Cln1^227–546^) degradation. This chimera presented a shorter Cln2 N‐terminal region, and the PESTa and PESTb of Cln1. Chimera 3 stability was similar to that of Cln1 and Cln2 (Fig. 4). The protein level of Chimera 3 increased in the *cdc53*,* cdc4* and *grr1* mutant cells (Fig. 6B). This is consistent with the translational shut‐offs experiment results since Chimera 3 was stabilized in all the mutant strains as compared to the wild‐type (Fig. 6C). Thus, as occurred for Cln2 and Chimera 2, Chimera 3 would be degraded by both SCF^Grr1^ and SCF^Cdc4^. This is apparently surprising because this chimera contained all the Cln1 sequences involved in cyclin degradation. However, its degradation pattern did not correspond to Cln1, but to Cln2. The only region of Cln2 that remained in Chimera 3 was the N‐terminal region from amino acids 1 to 224. Therefore this region, and not the PEST sequences, would be responsible for determining the degradation pattern of the cyclin allowing the cyclin to be degraded not only by SCF^Grr1^, but also by SCF^Cdc4^.

**Figure 7:**
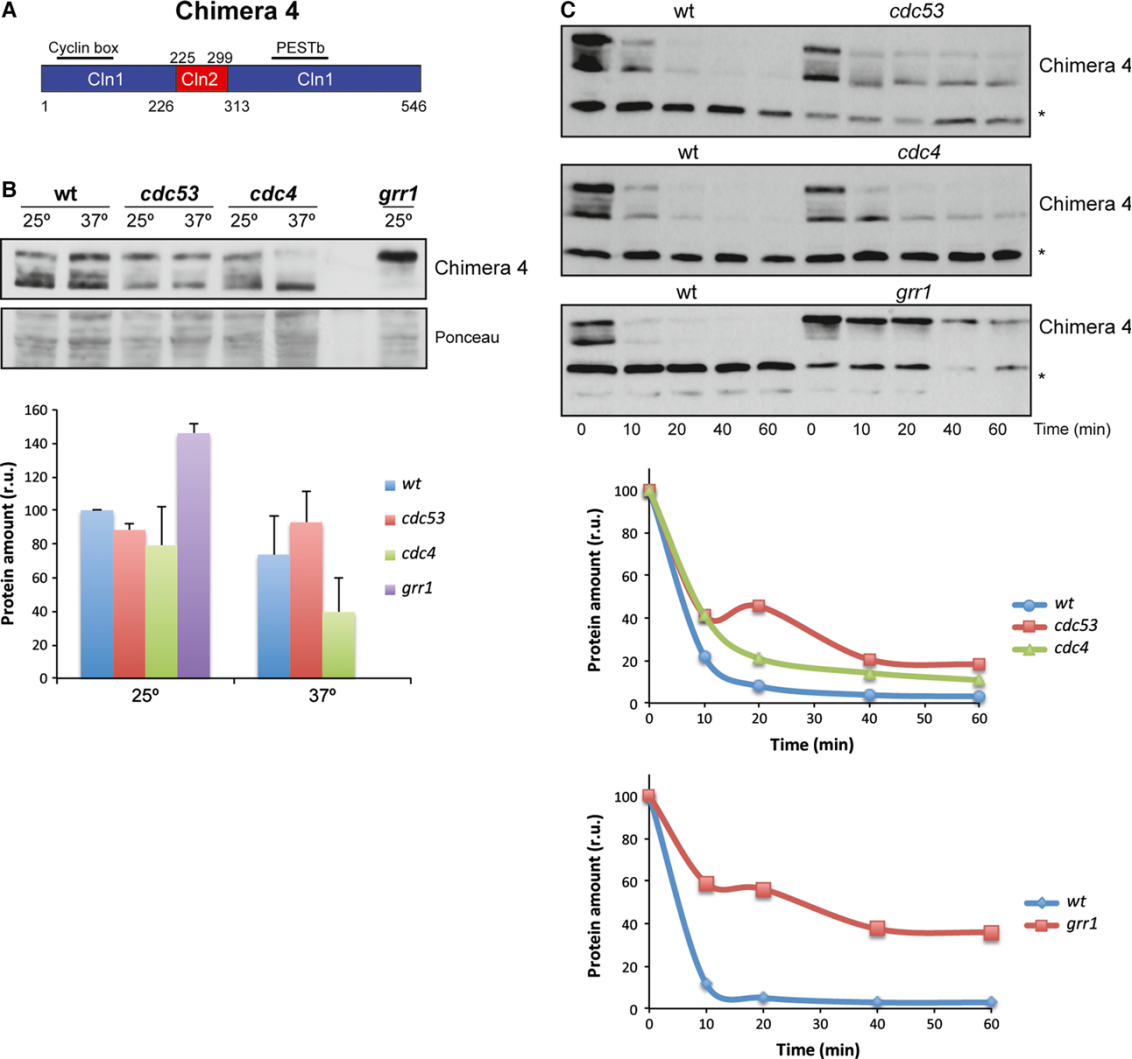
Analysis of Chimera 4 degradation in SCF ubiquitin‐ligase mutant strains. (A) Scheme of Chimera 4 cyclin. (B, C) Protein level and degradation of Chimera 4 cyclin were analyzed as described in Fig. 2. Ponceau staining or a nonspecific band labeled with an asterisk that cross‐react with the antibody is shown as loading control.

Finally, we used Chimera 4 (Cln1^1–226^‐Cln2^225–299^‐Cln1^313–546^), derived from Cln1 by replacing an internal fragment from the equivalent region of Cln2 (Fig. 7A). Consequently, Chimera 4 lost Cln1 PESTa, so it only contained Cln1 PESTb. The analysis of Chimera 4 level in the different degradation mutant strains indicated that the protein level was roughly similar to wild‐type cells in the case of mutant cells *cdc53* and *cdc4*. Conversely, Chimera 4 significantly increased in the absence of Grr1 (Fig. 7B). Once again, the translational shut‐off results were consistent with these observations since a significant stabilization of Chimera 4 was observed only in the case of the *grr1* mutant strain (Fig. 7C). All these observations were similar to that observed with Cln1 and allowed us to conclude that Chimera 4 was degraded, as Cln1, mostly by SCF^Grr1^. The fact that Chimera 4 followed a degradation pattern characteristic of Cln1, whereas Chimera 2 followed that of Cln2, further reinforced the conclusion that the N‐terminal end of the cyclin was somehow involved in the control of its degradation. More specifically, the region of Cln2 from amino acids 1 to 224 allowed SCF^Cdc4^ to control cyclin stability.

**Figure 8:**
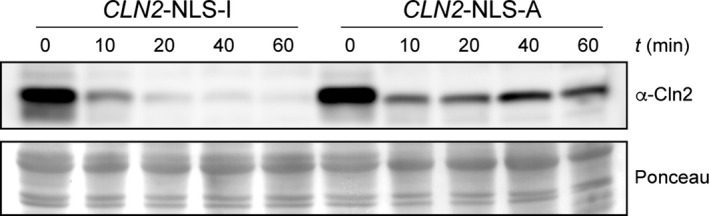
Protein stability of Cln2 protein with altered localization. Protein level of Cln2 cyclin fused to two copies of either active or inactive SV40 NLS was analyzed at the indicated time after the addition of cycloheximide by western blot. Ponceau staining is shown as loading control.

Furthermore, the analysis of the Chimera 4 degradation in wild‐type cells revealed that this cyclin was much more unstable than Cln1 (Fig. 4). The Cln2 fragment inserted in Chimera 4 contained a nuclear export signal 55. Hence, this result could indicate that cyclin stability might be affected by its subcellular location, so the nuclear export of the cyclin resulted in greater instability. To investigate this possibility, the stability of a version of Cln2 shifted to the nucleus due to the fusion of two copies of the SV40 nuclear localization signal 56, was analyzed. As it can be observed in Fig. 8, increasing Cln2 nuclear localization resulted in a significant stabilization of the protein. This result is consistent with the increased instability of Chimera 4 and strongly supports that these cyclins are more stable in the nucleus than in the cytosol.

## Discussion

Cyclins Cln1 and Cln2 accumulate at the end of G1 at the Start transition. This is critical for the cell cycle to start at the right time. Cyclins are constitutively unstable, therefore, the control of Cln1 and Cln2 stability is a key mechanism for the proper regulation of cell cycle progression. In almost all previous studies, Cln1 and Cln2 degradation has been studied without distinguishing between them or only Cln2 has been investigated. For this reason, we decided to analyze the degradation of Cln1 and Cln2 separately in order to establish whether there are differences between these cyclins in this regulatory mechanism. First, we compared the stability of the two cyclins. Previous studies using pulse‐chase or transcriptional shut‐off experiments have reported a half‐life for Cln2 or total Cln1,2 ranging between 5 and 15 min 20, 24, 25, 26, 27, 28, 29. In agreement with these values, we observed a half‐life of around 10 min for both Cln1 and Cln2 in the translational shut‐off assays. To our knowledge, this is the first time that the stability of endogenous Cln1 has been addressed independently of Cln2, and our results indicate that both cyclins show an identical stability.

PEST sequences have been described as determinants of the instability of proteins degraded via the SCF ubiquitin ligase 59, 60. In this work, we attempted to analyze the functionality of the PEST sequences present in Cln1 and Cln2, which proved especially relevant in the case of the PESTb of Cln1 given its low predictive value as a PEST. We conclude that Cln1 PESTb is functional and that, in fact, it seems to confer greater instability than the equivalent PEST of Cln2, as deduced from the shorter half‐life of Chimera 2 compared to Cln2. However, it cannot be ruled out that the greater instability of Chimera 2 is caused by adjacent regions to the PEST sequence, which may affect its functionality. Consistent with this, it has been described that removing or introducing PEST sequences to different cell cycle regulators affects their stability but the effects are magnified if neighboring domains are included. This is the case of the stable versions of Cln1, Cln2 and Cln3, which lose large areas covering all or part of the PEST regions 20, 61. Likewise, the transfer to a heterologous protein of the entire C‐terminal domain of Cln2 but not the transfer of just the PEST region has a quantitative important effect on protein stability 26, 31. So it is possible that regions around PESTb in the C‐terminal region of Cln1 may modulate its function. In any case, it is clear that the interchange of the C‐terminal region for that of Cln1 destabilizes the Cln2 protein. It must be note that this destabilization does not occur when a larger fragment from Cln1 is introduced (Chimera 3). This must be due to the absence of a nuclear export mechanism in Chimera 3 and to the influence of subcellular localization in the cyclin stability (see below).

Another issue addressed in this work is the analysis of the ubiquitin ligase involved in Cln1 and Cln2 degradation. It is widely accepted that both cyclins are degraded by SCF^Grr1^ upon the basis that Cln1 and Cln2 stability increases in *grr1* 20, 23, *cdc53* 24 and some *skp1* 62 mutants, and, in addition, Cln1 and Cln2 bind Grr1 *in vitro* 23, 30. We have shown that both Cln1 and Cln2 are, in fact, degraded by the SCF^Grr1^ pathway but, in addition, we conclude that the degradation of Cln2, but not of Cln1, also depends on the SCF^Cdc4^ pathway. As regards the involvement of Cdc4 in Cln degradation, the literature offers contradictory results: some authors report that there is no stabilization of Cln2 in a *cdc4* mutant strain, whereas others indicate a decrease in the Cln2 degradation rate in *cdc4* cells, although considerably slighter than the effect observed in a *grr1* mutant strain 23, 32, 33, 34. To support Cdc4's ability to mediate Cln2 degradation, Cdc4, which is normally located inside the nucleus, has been recently described to drive Cln2 degradation when forced to locate in the cytoplasm 23. This suggests that Cln2's susceptibility to Cdc4 must depend on the colocalization of both proteins, so the differences in the reported results may be due to differences in the localization of Cln2 in yeast cells under the experimental conditions used. Our results clearly indicate that Cln2 is stabilized in a *cdc4* mutant strain and, more importantly, that this is a specific behavior of Cln2 as compared to Cln1. Hence, this establishes a new regulatory difference between these two cyclins.

The results obtained with different chimeric cyclins can help to characterize the cyclin regions responsible for the differences observed between Cln1 and Cln2 degradation. Table 1 summarizes the significant sequences of each chimera and the results obtained in terms of the degradation pattern and stability. The fusion of the C‐terminal region of Cln2 (amino acids 376–545), including the PEST sequence and the adjacent regions, to a stable version of Sic1, allows recognition by SCF^Grr1^, but not by SCF^Cdc4^ 31. Moreover, the exchange of this region between Cln3 and Cln2, causes that Cln3 ubiquitination, which is normally mediated by both SCF^Grr1^ and SCF^Cdc4^, becomes specific of SCF^Grr1^ 23. Therefore, Cln2 PEST does not seem to mediate the specific effect of SCF^Cdc4^ in Cln2 degradation. In fact, when we exchanged Cln2 PEST for Cln1 PESTb (Chimeras 2 and 3), the degradation pattern remained unchanged, and both chimeras were still stabilized in the *cdc4* mutant strain. Thus, the PEST sequences of the two cyclins are equivalent in terms of recognition by the SCF complex, and another region of the protein must be responsible for determining sensitivity to SCF^Cdc4^. This region is the N‐terminal fragment of Cln2 (from 1 to 224) since is the only Cln2 sequence present in Chimera 3. This conclusion is reinforced by the results obtained with Chimera 4 and Chimera 2. They differ only in the N‐terminal region of the protein. Interestingly, Chimera 4, which contains the N‐terminal region corresponding to Cln1, shows the degradation pattern of Cln1, while Chimera 2, which contains the N‐terminal region corresponding to Cln2, shows the degradation pattern of Cln2. In conclusion, the N‐terminal region of the cyclin is responsible for the difference between Cln1 and Cln2 degradation as regards the involvement of SCF^Cdc4^.

**Table 1:** Cyclin properties

Cyclin	PEST	N‐terminal end	Nuclear export signal	F‐box prot	Stability
Cln2	PEST Cln2	Cln2	Cln2	Grr1 Cdc4	Standard
Cln1	PESTa Cln1 PESTb Cln1	Cln1	–	Grr1	Standard
Chimera 2	PESTb Cln1	Cln2	Cln2	Grr1 Cdc4	Less stability
Chimera 3	PESTa Cln1 PESTb Cln1	Cln2	–	Grr1 Cdc4	Standard
Chimera 4	PESTb Cln1	Cln1	Cln2	Grr1	Less stability

How can the N‐terminal region of the protein determine the degradation pattern of Cln1 and Cln2? Different possibilities are considered. The N‐terminal region contains the cyclin box, which is responsible for binding to Cdc28. Different observations suggest that Cdc28‐dependent phosphorylation is required for Cln2 degradation: the ubiquitination of Cln2 *in vitro* requires phosphorylation by Cdc28 29; inhibition of proteolysis during Start accumulates hyperphosphorylated forms of Cln2 20, 24; the mutation at Cdc28 phosphorylation sites stabilizes the Cln2 protein 28 and renders Cln2 unable to bind to Grr1 or Cdc4 23. For these reasons, the association with Cdc28 could influence cyclin degradation. It might be possible that differences in the cyclin‐Cdc28 binding mediated by the cyclin box explain the different degradation pattern of Cln1 and Cln2. However, the cyclin box is highly conserved between Cln1 and Cln2 (72% of identity), which argues against this possibility.

Another hypothesis is that some element present in the N‐terminal region is crucial for the recognition and degradation of the cyclin by SCF^Cdc4^. CPD have been described in most SCF^Cdc4^ substrates 22. In most of these cases, the importance of phosphorylation by Cdc28 at these recognition sites has been highlighted. We cannot rule out that similar sites in the N‐terminal region of Cln2 might be responsible for its recognition by SCF^Cdc4^; however, no consensus CPD sites exist in this region of Cln2, and Cdc28 phosphorylation sites are located in the C‐terminal region of the protein, thus ruling out this possibility.

Another possibility lies in the fact that the N‐terminal region of Cln1 and Cln2 contains the sequences that drive their nuclear import 55. Cdc4 is located mainly in the nucleus so recognition by SCF^Cdc4^ is conditioned by the subcellular localization of the target protein. Thus, Far1, which is ubiquitylated by SCF^Cdc4^, is stabilized when exported to the cytoplasm 17 or the differences between the Cln3 and Cln2 degradation patterns, as regards Cdc4 sensitivity, could be due to differences in their subcellular localization 23. The sensitivity of Cln2 but not of Cln1 to Cdc4 may, therefore, reflect that a significant amount of Cln2 degradation, but not of Cln1 degradation, is achieved inside the nucleus. When considering that the N‐terminal regions containing the nuclear import signals are responsible for Cdc4 sensitivity, it may be suggested that a different efficiency in nuclear import may determine the degradation pattern of cyclins Cln1 and Cln2.

The subcellular localization of the cyclin may have a greater influence on cyclin degradation aside from mediating the susceptibility to Cdc4. Chimera 4 is much more unstable than Cln1. This is apparently surprising because Chimera 4, in comparison with Cln1, lacks PESTa and only contains PESTb. Interestingly, Chimera 4 contains the Cln2 nuclear export signal, which actually mediates the nuclear export of Chimera 4, a mechanism that is absent in Cln1. Therefore, the nuclear export of the cyclin causes greater instability. Consistently with this idea, the greater instability of Chimera 2, as compared to Chimera 3, is lost when the Cln2 export signal present in Chimera 2 is eliminated in Chimera 3. More importantly, we have observed that forcing nuclear localization of Cln2 renders the protein more stable. All these observations allow us to propose that cyclins Cln1 and Cln2 are more unstable in the cytosol.

In this work, we have established a new regulatory difference between Cln1 and Cln2, traditionally considered equivalent cyclins. This reinforces the high degree of specialization between cyclins in eukaryotic cells.

## Author contributions

IQ acquired the data; IQ and JCI conceived and designed the project, analyzed and interpreted the data, and wrote the paper.
